# Ultrasound Determination of Gestational Age Using Placental Thickness in Female Dogs: An Experimental Study

**DOI:** 10.1155/2012/850867

**Published:** 2012-07-10

**Authors:** André Luiz Louzada Maldonado, Edward Araujo Júnior, Débora Sartori Mendonça, Luciano Marcondes Machado Nardozza, Antonio Fernandes Moron, Sérgio Aron Ajzen

**Affiliations:** ^1^Department of Imaging Diagnostic, Federal University of São Paulo (UNIFESP), 05303-000 São Paulo, SP, Brazil; ^2^Department of Obstetrics, Federal University of São Paulo (UNIFESP), 05303-000 São Paulo, SP, Brazil

## Abstract

*Objective*. To verify if the placental thickness allows determining the gestational age, evaluating the correlation between the referred gestational age with the studied one, and the accuracy of the placental thickness measurement (biometry) with fetal morphologic parameters in bitches. *Methods*. The placental thickness of 336 bitches of diverse breeds was evaluated. Bitches were divided in three groups by body weight: small, medium, and big large size. The gestations pregnancies were evaluated by ultrasound from the third week of gestation. An analysis was performed between the mean values of the gestational age obtained of placental thickness by adjustment of curves and the reported gestational age. Student's *t*-test was applied to compare the mean of reported and placental thickness gestational age. Significance was defined as *P* < 0.05. *Results*. A positive and statistically significant correlation exists between the placental thickness and gestational age. The expression that presents the best correlation coefficient and explanation was thickness of placenta = 0.021*x* gestational age −0.314. *Conclusion*. It is possible to determine the gestational age in relation to the placental thickness measured by ultrasound in bitches with a satisfactory accuracy in relation to fetal morphologic parameters as gestational vesicle, ribs, or kidneys.

## 1. Introduction

Ultrasound in veterinary medicine contributes to the diagnosis of several diseases in both small and large size animals [1, 2]. The major advantage of the ultrasound examination is the possibility of evaluating the internal architecture and the structure of the parenchyma of abdominal organs. Ultrasound is the best method for the diagnosis of pregnancy and for the evaluation of fetal viability. In addition, it is the most sensitive and specific method in the evaluation of gestational age [3, 4]. Although there are numerous works correlating the size and the gestational age by mathematic formulas based on biparietal and abdominal diameters and on the size of the gestational sac, the simplest way to the determine the approximate age of the fetus is by observing the presence of some anatomic structures [5, 6]. However, these studies do not make reference to the measurement of the placenta as a method for the determination of gestational age. 

The placenta is the first fetal organ to develop and has primordial and critical functions. It mediates implantation and establishes an interface for the exchange of nutrients and gas in the maternal-fetal circulation, affecting the local immunological mediators, the maternal cardiovascular system, and metabolic functions [7]. The thickness of the placenta may assist in the diagnosis of gestational age in large bitches. 

The objective of this study was to verify if the evaluation of placental thickness by ultrasound correlates with gestational age in bitches of different breeds and sizes and to evaluate preciseness of the measurement of placental thickness (biometry) with fetal morphological parameters.

## 2. Methods

A cross-sectional and observational study was conducted in 336 pregnant bitches of several breeds, ages, and parity. The present study was approved by Federal University of São Paulo (UNIFESP) Ethics Committee. Bitches were divided into three groups, based on body weight: small, medium, and large sized according to standards established by the International Cynological Federation (*Fédération Cynologique Internationale*). Pregnancies were evaluated by ultrasound examination after the third week of gestation. Each bitch was evaluated once. All examinations were preceded by clipping the hair from the umbilical scar to the pubis, and by fastening the animals for at least four hours. Bitches were evaluated in dorsal, right and left lateral recumbence. 

**Figure 1:**
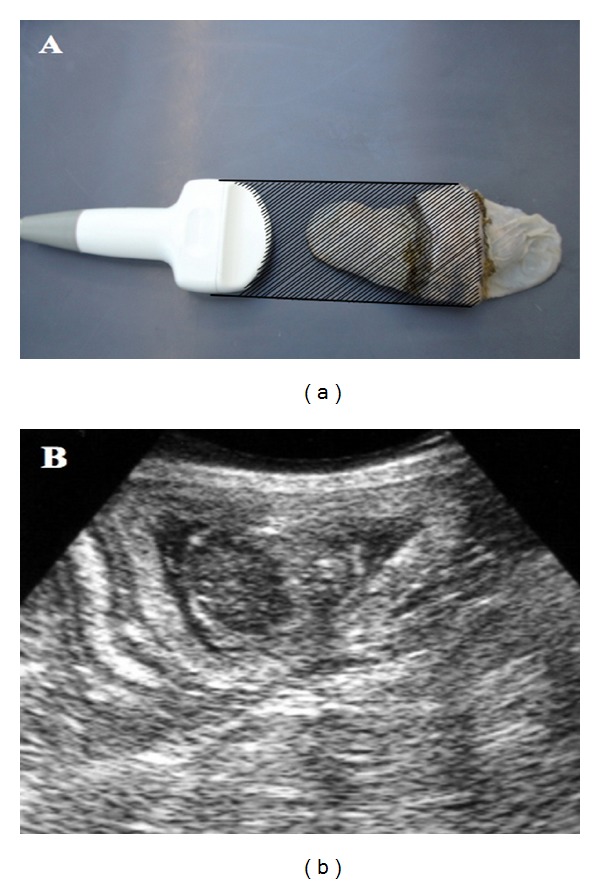
Planimetric image of the ultrasound beam's action angle in the placenta ring. The hachured area represents the angle (approximately 90°) of the beam's incidence on the placenta ring pars intermedia, the point where thickness measurements were performed (a). (b) Corresponding ultrasound image.

Ultrasound assessment of the placenta was performed in a longitudinal view perpendicular to the placenta's plane, next to its central area, measuring the outer layer (Figure 1). A EUB-405 (Hitachi, Tokyo, Japan) ultrasound unit was used as equipment, with a convex 5 MHz and/or linear 7.5 MHz probes. Images were captured using a Sony 890 md printer, with Sony brand thermo sensible paper model UPP-110S High Quality Printing Paper Type I (normal).

Placental thickness evaluation was divided into three stages. First, the linear expression of curves adjustment (*y* = 0.021*x* − 0.314 [8]) was used, in which “*y*” represents the thickness (cm) and “*x*” the gestational age (days). The linear expression was applied for all measurements in the three body weight groups and was correlated with the gestational age reported by owners. Next, the expression of curves adjustment was performed to explain the relationship between the gestational age reported by animals' owners and the age estimated from the placental thickness. Lastly, all expressions were compared among themselves and with their relationships were performed using curves adjustment to best explain the relationship between the reported and evaluated gestational age according to the placental thickness for all breeds. For this purpose, in our casuistic were included placental thickness measurements of the giant breed [8]: 22 Great Dane bitches, evaluated by ultrasound examinations in 7 days intervals after the third week of pregnancy, in a total of 88 measurements.

Pearson's correlation coefficient (*r*) with a scatter diagram was used to verify the existence and/or to characterize the relationship between gestational age and placental thickness for the three body weight groups. Curves adjustment was used in order to check which expression that best explained the relationship between reported and placental thickness obtained by ultrasound gestational age. Student's *t*-test was applied for the comparison between gestational age averages considering placental thickness and the reported gestational age for all study stages. The level of significance was *P* < 0.05.

## 3. Results

**Figure 2:**
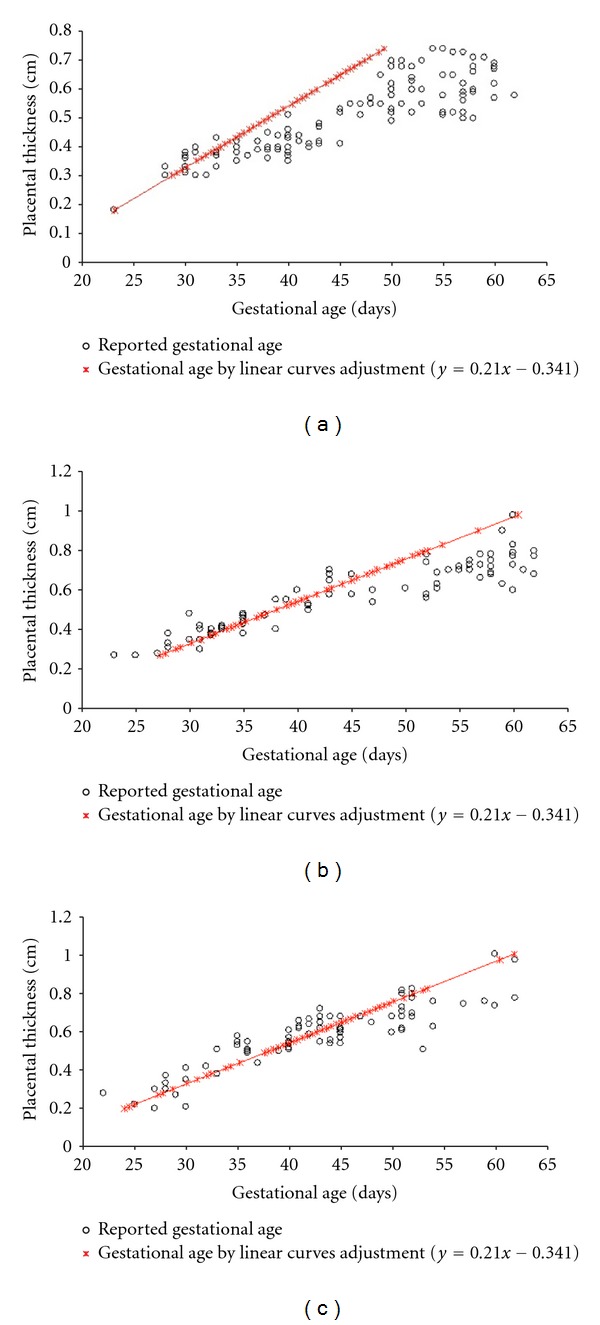
Scatter plot of the relationship of gestational age reported by owners and the one calculated by the linear expression *y* = 0.021*x* − 0.314 to small (a), medium (b), and large (c) size breed.

Placental thickness of 336 bitches were evaluated, of which 138 (41%) were of small size breeds, 108 (32%) of medium size breeds, and 90 (27%) of large size breeds. The relationship between gestational age reported by owners and gestational age calculated by the linear expression *y* = 0.021*x* − 0.314 is shown in Figure 2. The comparison between average gestational age determined by the linear curves adjustment and the reported gestational is shown in Table 1. There was a significant relationship between gestational age and the reported gestational age. The relationships between curve adjustments (linear, logarithmic, potential, and exponential) for placental thickness and reported gestational age values for the small, medium, and large breeds were, respectively: *y* = 0.0087*x* ^1.0637^, *R* ^2^ = 0.82, *P* < 0.01; *y* = 0.0109*x* ^1.0412^, *R* ^2^ = 0.87, *P* < 0.01; *y* = 0.0153*x* − 0.0737, *R* ^2^ = 0.80, *P* < 0.01, where “*y*” represented the thickness (cm) and “*x*” the gestational age (days).

**Figure 3:**
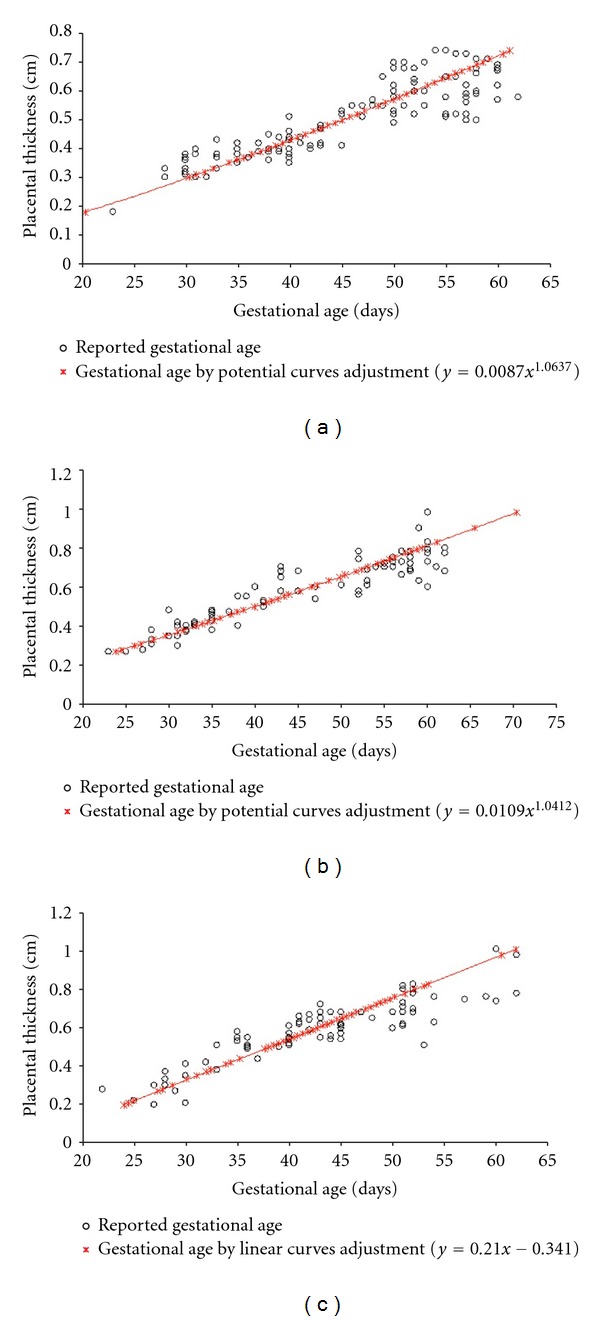
Scatter plot of the correlation between the gestational age defined by the potential adjustment and the reported age for the small (*y* = 0.0087*x* ^1.0637^—(a)), medium (*y* = 0.0109*x* ^1.0412^—(b)), and large (*y* = 0.0153*x* − 0.0737—(c)) size breed.

**Figure 4:**
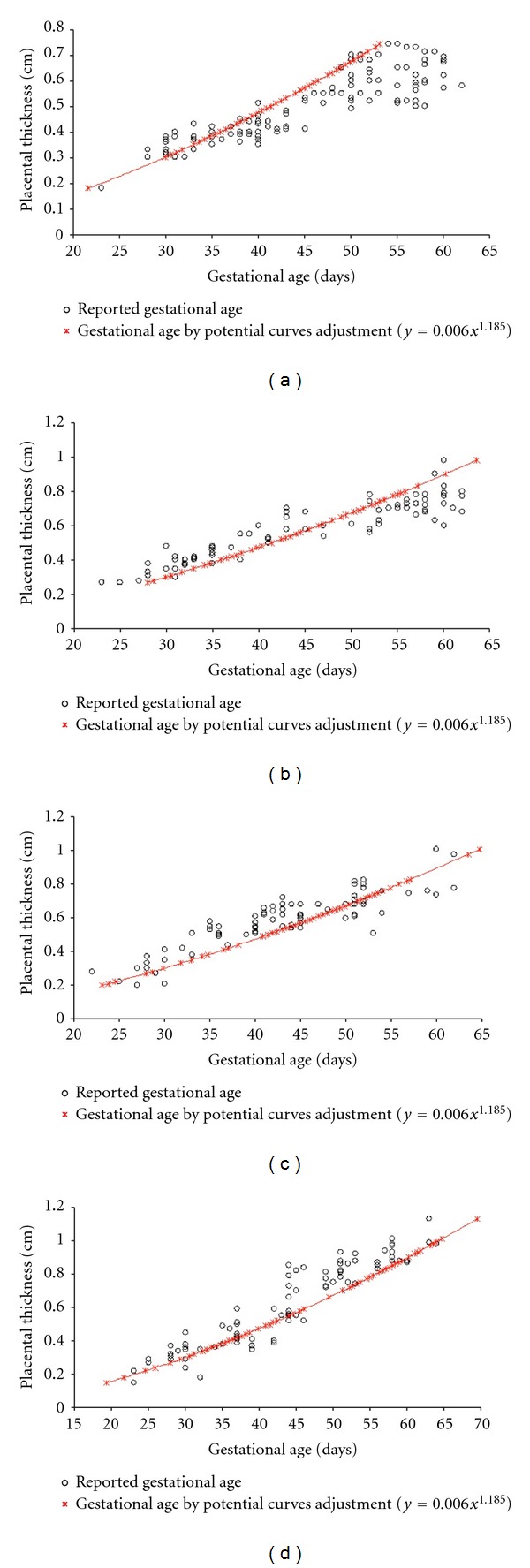
Scatter plot of the correlation between the gestational age defined by the potential adjustment and the reported age for the small (*y* = 0.006*x* ^1.185^—(a)), medium (*y* = 0.006*x*1.185—(b)), large (*y* = 0.006*x* ^1.185^—(c)), and giant (*y* = 0.006*x* ^1.185^—(d)) size breed.

The relationship between gestational age calculated by the expression of the curve adjustment for small, medium, and large breeds is shown in Figure 3. The comparison between the average gestational age determined by curves adjustment and the reported gestational age is shown in Table 2. There was no significant difference between the average reported gestational age and the one calculated by curves adjustment. From 424 measurements, the relationships between curve adjustment performed for placental thickness and reported to gestational age for the small, medium, large, and giant breed was *y* = 0.006*x* ^1.185^, *R* ^2^ = 0.75, *P* < 0.01 where “*y*” represents the thickness (cm) and “*x*” the gestational age (days). The relationship between gestational age calculated by the expression of curve adjustment and reported gestational age is shown in Figure 4. Comparisons between expressions of curves adjustment developed with regard to the placental thickness for small, medium, large, and giant size breeds is shown in Table 3. There was a significantly positive correlation between the placental thickness and the gestational age (*y* = 0.021*x* − 0.314).

## 4. Discussion

This study verified that the linear curves adjustments are applicable to bitches of different breeds and sizes [8]. Placental thickness is a significant parameter in the determination of gestational age, using the linear expression *y* = 0.021*x* − 0.314. The majority of bitches used in this study had large litters, which allowed for several measurements to be performed per bitch and for observation of homogeneity within each litter, with regard to the evaluated placental thickness. In bitches with more than eight puppies, fetal measurements were difficult from the hypogastric and epigastric regions at the end of gestation. In these cases, only fetuses that were positioned so they could be evaluated were included.

A simple regression is based on a linear relationship between the dependent variable (placental thickness) and the independent variable (gestational age). When the relationship is nonlinear (as was the case for small breeds), the independent variables needed to be transformed to render the relationship linear (*y* = 0.021*x* − 0.314). Parameters usually studied within the period from 20 to 30 days are gestational vesicles (of a necklace appearance), trabecula, fetus adhered to the dorsal gestational sac, fetal limits and heart rate and jaw mineralization [5, 6]. Our findings show that within such window, placental thickness would measure an average of 0.34 cm and, therefore, would refer to 30.5 days of pregnancy, according to the linear equation. Within the period from 30 to 40 days, parameters are visualization of ribs, thoracic, lumbar and cervical spine and head [5, 6]. Our findings show that within such window, placental thickness would measure an average of 0.44 cm and, therefore, would refer to 35.2 days of pregnancy, according to the linear equation. Kidneys, lungs, liver, differentiation between kidney cortex and medulla are seen with 40 to 50 days [5, 6]. Our findings show that within such window, placental thickness would measure an average of 0.67 cm and, therefore, would refer to 46 days of pregnancy, according to the linear equation. Within the period from 50 to 60 days, it is possible to see the following structures: stomach, urinary bladder and gallbladder, gastrointestinal tract motility, stomach and urinary bladder filling and decreased heart rate [5, 6]. Our findings show that within such window, placental thickness would measure an average of 0.89 cm and, therefore, would refer to 56.3 days of pregnancy, according to the linear equation.

An unexpected finding during this study was placental calcification. Placental calcification has been reported in rats. Dense calcium deposits were found in the maternal-fetal relationship before the chorion-allantoid placenta was formed, and additional deposits developed in placenta girdle during late gestation [9]. Placental calcification has also been reported in humans [10], where diabetic women's placentas are generally much more calcified [11]. A relationship between hyperglycemia and placental calcification could not be determined because a very low number of bitches had hyperglycemia.

In human medicine, evaluation of biparietal diameter is very effective in the beginning of pregnancy; however, as it develops, values keep losing their efficacy [12]. We compared biparietal diameter in English Bulldogs and Beagles which both were very close heights at withers; but their body masses are very different. In this comparison, the fetal measurement was highly imperfect for the diagnosis of gestational age; dogs show a very huge disparity in this parameter [13–15]. 

We also compared Great Danes, which have very long femurs, to Dachshund or Teckel, a chondrodystrophic breed. In this comparison, differences of more than one centimeter in the length were observed in fetuses of the same gestational age. Such difference would demonstrate that the use of such parameter is unfeasible. It is important to note that an operator's lack of experience would result in erroneous measurements [16]. Also, a nonexperienced examiner may face some difficulties in differentiating the end of the uterus wall and the beginning of the placenta, even with good quality equipment. The ultrasound view must be made at a right angle, or it can lead to an increase or decrease in placental thickness. In pregnancies with a very high number of puppies, a measurement of all placentas is unfeasible. Low resolution equipment may also result in erroneous measurements [16].

In summary, it is possible to determine the gestational age with regard to placental thickness measured by ultrasound in bitches of different breeds and sizes. Evaluation of gestational age by placental biometry showed satisfactory precisions with regard to fetal morphological parameters.

## Figures and Tables

**Table 1:** Comparison between gestational age and reported age averages for the three evaluated breeds and statistical analysis.

Breed	*n*	Average		Standard deviation		Average standard error		*P* value
		RGA	GA	RGA	GA	RGA	GA
Small size	138	45.78	38.49	9.94	6.09	0.85	0.52	<0.001^∗^
Medium size	108	45.10	41.62	11.12	7.22	1.07	0.69	<0.001^∗^
Large size	90	42.74	41.76	9.24	7.37	0.97	0.78	0.030^∗^

*n*: number of female dog, RGA: reported gestational age, GA: gestational age obtained considering the curves adjustment.

^∗^Student's *t*-test.

**Table 2:** Difference between gestational ages calculated by curves adjustments and reported age averages for small, medium, and large size breeds and statistical analysis.

Breed	*n*	Average		Standard deviation		Average standard error		*P* value^∗^
		RGA	GA	RGA	GA	RGA	GA
Small size	138	45.78	45.59	9.94	9.19	0.85	0.78	0.617
Medium size	108	45.10	44.90	11.12	10.25	1.07	0.99	0.642
Large size	90	42.74	42.81	9.24	10.36	0.97	1.09	0.890

*n*: number of female dogs, RGA: reported gestational age, GA: gestational age obtained considering the curves adjustment.

^∗^Student's *t*-test.

**Table 3:** Comparison between expressions of curves adjustment determined with regard to placenta thickness for the studied breeds.

Breed	Curves adjustment								
	Individual^(a)^			*y* = 0.021*x* − 0.314			*y* = 0.006*x* ^1.185^		
	*r*	*R* ^2^	*P *value	*r*	*R* ^2^	*P* value	*r*	*R* ^2^	*P *value
Small size	0.895	0.828	<0.001^∗^	0.891	0.884	<0.001^∗^	0.898	0.756	<0.001^∗^
Medium size	0.922	0.876	<0.001^∗^	0.921	0.884	<0.001^∗^	0.923	0.756	<0.001^∗^
Large size	0.893	0.809	<0.001^∗^	0.893	0.884	<0.001^∗^	0.889	0.756	<0.001^∗^
Giant size	0.940	0.884	<0.001^∗^	0.940	0.884	<0.001^∗^	0.939	0.756	<0.001^∗^

^(a)^ *y* = 0.0087*x* ^1.0637^ for the small size breed, *y* = 0.0109*x* ^1.0412^ for the medium size breed, *y* = 0.0153*x* − 0.0737 for the large size breed, *y* = 0.021*x* − 0.314 for the giant size breed, *r*: Pearson's correlation coefficient, *R* ^2^: determination coefficient.

^∗^Student's *t*-test.
